# BPA-Induced Deregulation Of Epigenetic Patterns: Effects On Female Zebrafish Reproduction

**DOI:** 10.1038/srep21982

**Published:** 2016-02-25

**Authors:** Stefania Santangeli, Francesca Maradonna, Giorgia Gioacchini, Gilda Cobellis, Chiara Carla Piccinetti, Luisa Dalla Valle, Oliana Carnevali

**Affiliations:** 1 Dipartimento Scienze della Vita e dell’Ambiente, Università Politecnica delle Marche, Via Brecce Bianche, 60131 Ancona, Italy; 2 Dipartimento di Medicina Sperimentale, Seconda Università degli Studi di Napoli, Via S. Maria di Costantinopoli 16, 80138 Napoli, Italy; 3 Dipartimento di Biologia, Università di Padova, Via Ugo Bassi 58/B, 35131 Padova, Italy; 4 INBB Consorzio Interuniversitario di Biosistemi e Biostrutture, 00136 Roma, Italy

## Abstract

Bisphenol A (BPA) is one of the commonest Endocrine Disruptor Compounds worldwide. It interferes with vertebrate reproduction, possibly by inducing deregulation of epigenetic mechanisms. To determine its effects on female reproductive physiology and investigate whether changes in the expression levels of genes related to reproduction are caused by histone modifications, BPA concentrations consistent with environmental exposure were administered to zebrafish for three weeks. Effects on oocyte growth and maturation, autophagy and apoptosis processes, histone modifications, and DNA methylation were assessed by Real-Time PCR (qPCR), histology, and chromatin immunoprecipitation combined with qPCR analysis (ChIP-qPCR). The results showed that 5 μg/L BPA down-regulated oocyte maturation-promoting signals, likely through changes in the chromatin structure mediated by histone modifications, and promoted apoptosis in mature follicles. These data indicate that the negative effects of BPA on the female reproductive system may be due to its upstream ability to deregulate epigenetic mechanism.

Endocrine Disruptor Compounds (EDCs) are chemicals that can interfere with vertebrate reproduction^1^. The vertebrate reproductive system is under the control of the hypothalamic-pituitary-gonadal axis, which operates through a hormonal cascade where production of gonadotropin-releasing hormone (GnRH) in the hypothalamus stimulates pituitary gonadotropin synthesis, eventually leading to production of steroid hormones by the gonads.

Bisphenol A (BPA) is commonly used in the manufacturing of polycarbonate plastics and epoxy resins, making it one of the most widespread EDCs worldwide. According to recent estimates, 100 ton of BPA per year may end up in the environment^2^ due to landfill or plastic degradation^3^, resulting in concentrations of 5 μg/L to 21 μg/L in environmental water^4^.

BPA alters gene expression by binding to nuclear estrogen receptors (ERs) ERα and ERβ. It is defined as a selective ER modulator because its effects are pro-estrogenic in some tissues and anti-estrogenic in others^5^,^6^. Its ability to bind to membrane receptors makes it harmful even at pico- and nanomolar concentrations^6^.

As an environmental pollutant, BPA is probably able to affect epigenetic mechanisms through methylation of CpG sites^7^. It also seems to induce histone modification, altering the chromatin structure and through it transcription activation and repression^5^.

The first goal of the present study was to determine the effects of BPA concentrations consistent with environmental exposure (5, 10, and 20 μg/L) on reproductive physiology in female vertebrates. To do this, the expression of several genes critical for reproduction was monitored in the ovary: ERs *esr1* and *esr2a*, steroidogenic acute regulatory protein (*star*), and a member of the cytochrome P450 family 11 (*cyp11a1*)^8^,^9^,^10^,^11^ for follicle development and steroidogenesis; progesterone receptors *pgrmc1* and *pgrmc2* for oocyte growth and maturation; and bone morphogenetic protein 15 (*bmp15*) and growth differentiation factor 9 (*gdf9*) as local signals^12^. Apoptosis, autophagy, and oocyte viability were also assessed.

The second goal of the study was to explore whether the changes in the expression levels of these genes were caused by histone modifications, as measured by lysine 4 and 27 trimethylation in the amino terminal of histone 3 (respectively H3K4me3 and H3K27me3). H3K4me3 is involved in the activation of transcription^13^, since it marks the Transcription Start Site (TSS) region, whereas H3K27me3 is chiefly associated with gene silencing^14^. Based on the recent hypothesis of a possible interplay between histone modifications and DNA methylation patterns, where DNA methyltransferases (DNMTs) seem to play an important role^15^, DNMT gene expression levels were also investigated.

The experimental model selected for the study, *Danio rerio*, has a fully sequenced genome and shares with humans 70% gene orthology and body plan similarity^16^; these features make it an ideal model to assess the effects of EDCs on human reproduction. The issue is both urgent and topical, since several studies have reported a decline in human fertility, especially in western countries^17^,^18^. Even though the decrease may be related to a variety of factors, a role for environmental EDCs is strongly suspected^1^.

## Results

### Fertility

Fertility was measured as mean number ± standard deviation (SD) of fertilized eggs per female per day from the 8^th^ to the 21^st^ day of BPA treatment. The results showed blocked ovulation (0 ± 0 eggs) 8 days into treatment in fish receiving the lowest BPA concentration (5 μg/L), whereas the intermediate (10 μg/L) and the highest (20 μg/L) concentration did not induce significant changes (respectively 314 ± 8 and 330 ± 9 eggs) compared with control fish (333 ± 12 eggs).

### Molecular analysis of the genes related to oogenesis

The lowest of the 3 concentrations tested, 5 μg/L, induced the most severe effects. Molecular analysis demonstrated that BPA can inhibit reproduction, particularly by affecting oocyte maturation.

**Figure 1:**
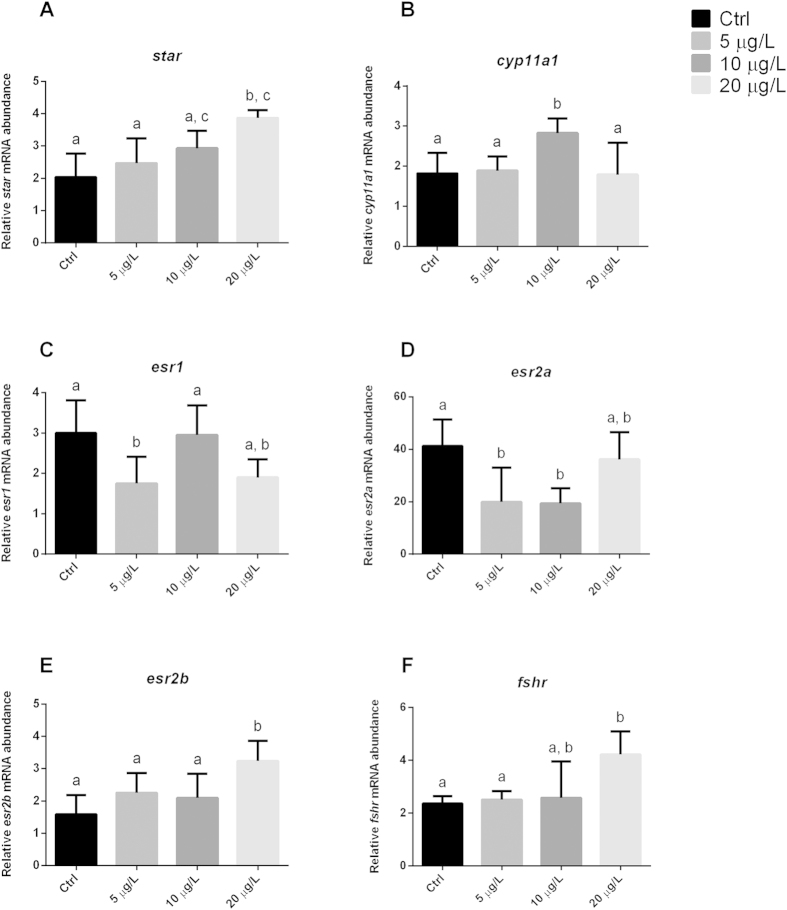
Transcription profiles of genes involved in steroidogenesis and oocyte growth. Letters above each column indicate statistical differences among groups (*p < *0.05 vs. untreated controls; ANOVA followed by Tukey’s multiple comparison test). (**A**) *star*: steroidogenic acute regulatory protein (F = 6.710; P = 0.0049); (**B**) *cyp11a*: cytochrome P450, family 11, subfamily (F = 4.608; P = 0.0178); (**C**) *esr1*: estrogen receptor 1 (F = 5.192; P = 0.0099); (**D**) *esr2a*: estrogen receptor 2a (F = 5.350; P = 0.0115); (**E**) *esr2b*: estrogen receptor 2b (F = 5.890; P = 0.0066); (**F**) *fshr*: follicle stimulating hormone receptor (F = 4.586; P = 0.0257). Data were generated in duplicate from five biological replicates.

**Figure 2:**
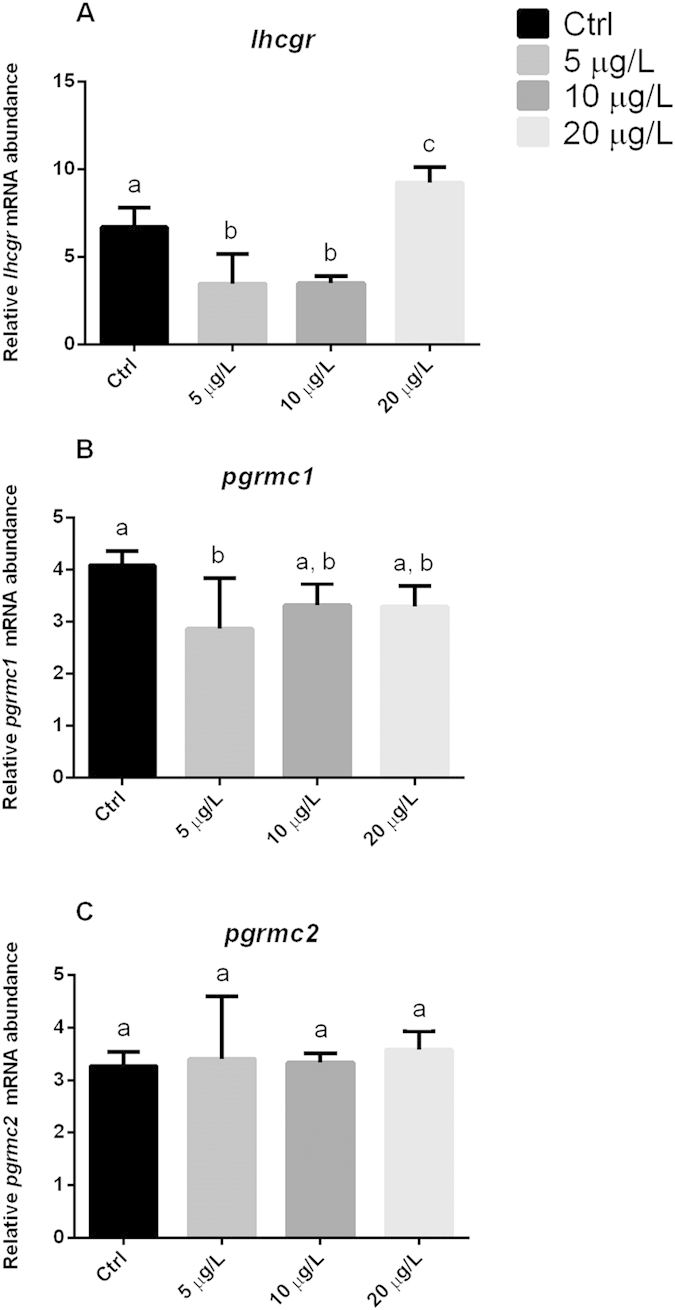
Transcription profiles of genes involved in oocyte maturation and germinal vesicle breakdown. Letters above each column indicate statistical differences among groups (*p < *0.05 vs. untreated controls; ANOVA followed by Tukey’s multiple comparison test). (**A**) *lhcgr*: luteinizing hormone/choriogonadotropin receptor (F = 29.18; P = 0.0001); (**B**) *pgrmc1*: progesterone membrane receptor component 1 (F = 3.382; P = 0.0462); (**C**) *pgrmc2*: progesterone membrane receptor component 2 (F = 0.1755; P = 0.9114). Data were generated in duplicate from five biological replicates.

The lowest concentration induced significant downregulation of *esr1* and *esr2a* (Fig. 1C,D), whereas it did not significantly affect the transcription pattern of the other genes involved in steroidogenesis and oocyte growth, *esr2b*, *star*, *cyp11a1*, and follicle stimulating hormone receptor (*fshr*) (Fig. 1A,B,E,F). It also significantly reduced the transcripts of genes related to oocyte maturation and germinal vesicle breakdown processes (i.e. luteinizing hormone/choriogonadotropin receptor, *lhcgr*, and *pgrmc1*) (Fig. 2A,B), whereas there was no change in *pgrmc2* expression (Fig. 2C).

The 10 μg/L concentration significantly up-regulated the *cyp11a1* gene (Fig.1B) and significantly down-regulated *esr2a* and *lhcgr* (Figs 1D and 2A), whereas the remaining genes were not affected.

The highest BPA concentration, 20 μg/L, positively and significantly interfered with the expression of *esr2b*, *star*, and *fshr*, the genes involved in steroidogenesis and oocyte growth (Fig. 1A,E,F), and of *lhcgr*, which has a role in oocyte maturation (Fig. 2A). Such changes were associated with a complete block of reproduction 8 days into treatment with the lowest BPA concentration, whereas no changes in fecundity were seen in the other groups.

Importantly, none of the BPA concentrations tested affected the expression of *bmp15* and *gdf9* (Table 1), which are considered as local signals.

The expression trends of autophagy/beclin-1 regulator 1a (*ambra1a*) and *beclin1* (Table 1), two key signals involved in the autophagy process, were not affected. In contrast, apoptosis gene expression exhibited a differential pattern, where the effector *caspase3* (Table 1) was up-regulated by all 3 concentrations, while tumor protein 53 (*tp53*) (Table 1) was up-regulated only by the lowest.

### Histological analysis

Oocyte viability was assessed by histology, to document the effects of 5 μg/L BPA on the ovary.

**Figure 3:**
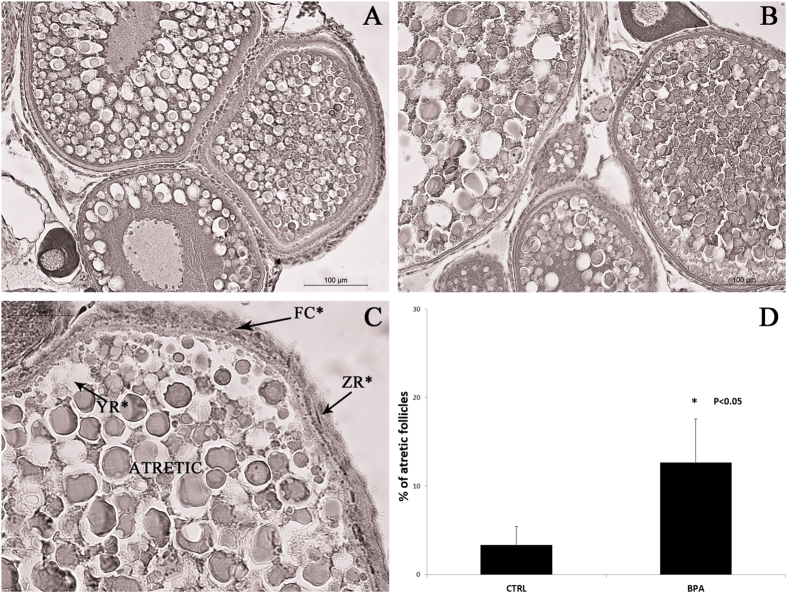
Histological analysis of ovaries from control fish (**A**) and fish exposed (**B**) to 5 μg/L BPA. Ovarian sections show different follicular stages and atretic follicles. (**C**) A follicle with the morphological markers of atresia: ZR*: Zona radiata breakdown; YR*: Yolk resorption; FC*: Follicular cell proliferation. (**D**) Percentage of atretic follicles in ovary from control fish (CTRL) and fish exposed (BPA) to 5 μg/L BPA.

Findings showed that total follicle number did not change in any experimental group, and that pre-vitellogenic follicles grew normally and developed into vitellogenic and mature ones. However, nearly all exposed mature follicles exhibited morphological evidence of atresia, including membrane disintegration, follicle cell proliferation, zona radiata breakdown, and yolk resorption (Fig. 3).

### ChIP-qPCR analysis

To establish whether the gene expression alterations caused by exposure to 5 μg/L BPA could be caused by epigenetic changes, H3K4me3 and H3K27me3 enrichment was assessed in the TSS region, in its two flanking regions, and in other gene body regions by evaluating *star*, *fshr* and *lhcgr*, which are related to steroidogenesis, oocyte growth, and oocyte maturation.

**Figure 4:**
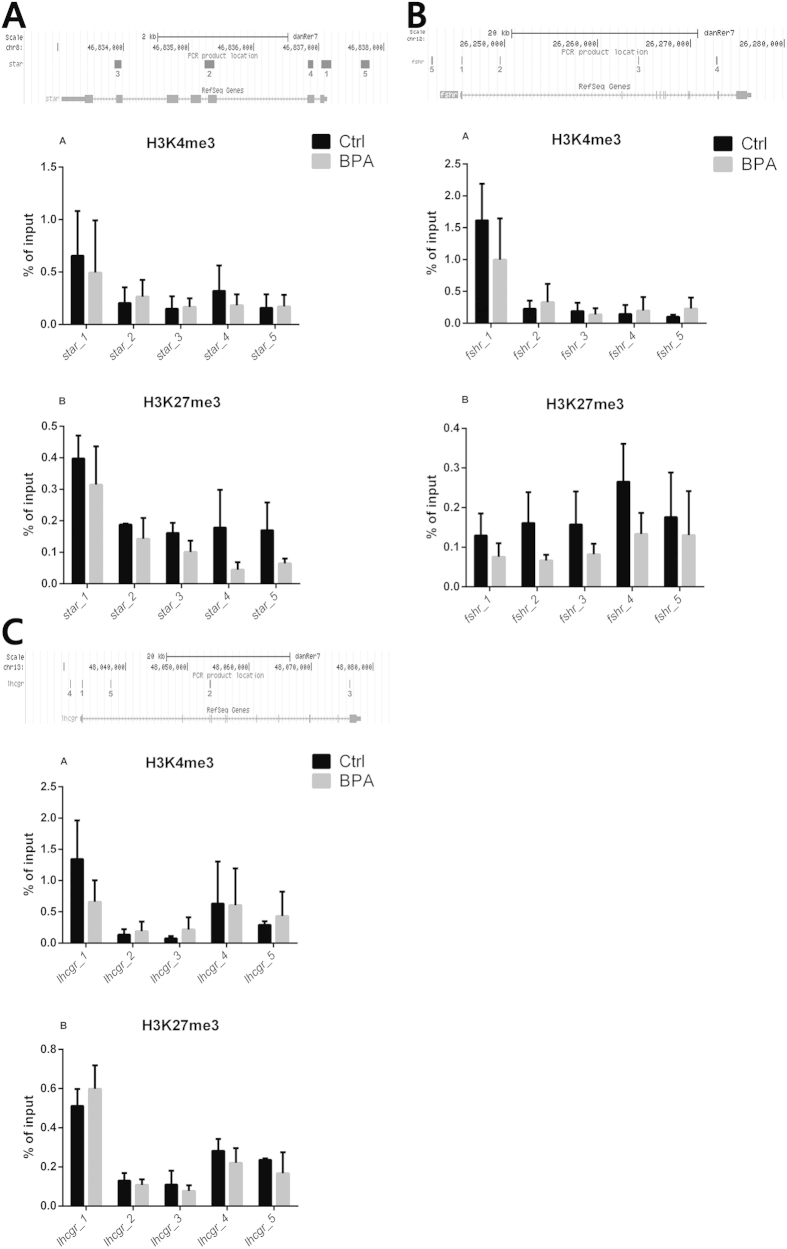
ChIP-qPCR analysis of H3K4me3 and H3K27me3 trimethylation of lysine 4 (K4) and 27 (K27) in the amino terminal of histone 3 (H3) enrichment. (**A**) ChIP-qPCR results for *star* (steroidogenic acute regulatory protein) gene associated with H3K4me3 and H3K27me. (**B**) ChIP-qPCR results for *fshr* (follicle stimulating hormone receptor) gene associated with H3K4me3 and H3K27me. (**C**) ChIP-qPCR results for *lhcgr* (luteinizing hormone/choriogonadotropin receptor) gene associated with H3K4me3 and H3K27me.

H3K4me3 enrichment was reduced in the TSS and the *star*_4 region (Fig. 4A) of BPA-treated ovaries compared with control ovaries, whereas it was unchanged in the remaining regions (Fig. 4A). Similarly, H3K4me3 enrichment in the *fshr* TSS region was reduced in BPA-exposed compared with control ovaries and was unaffected in the other regions (Fig. 4B). Notably, the H3K27me3 epigenetic marks for *star* and *fshr* were weaker in treated than in control ovaries at all sites (Fig. 4A,B), reflecting similar trends in H3K4me3 and H3K27me3 histone modifications. As regards oocyte maturation, the H3K4me3 mark appeared to be fainter in the *lhcgr* TSS region of treated compared with control ovaries, whereas no changes were detected in the other regions (Fig. 4C). In contrast, increased H3K27me3 enrichment was documented in the TSS gene of treated compared with control fish (Fig. 4C). In addition, reduced and similar levels of H3K27me3 enrichment were found in the flanking regions and in the remaining regions of the TSS, respectively (Fig. 4C).

### Molecular analysis of DNMTs

The expression of the DNMT isoforms involved in methylation maintenance, *dnmt1*, and *de novo* methylation, *dnmt3*, was significantly up-regulated in BPA-exposed compared with control ovaries (Table 2), whereas a reduction in the relative abundance of *dnmt4*, *dnmt6* and *dnmt7* transcripts was detected in treated ovaries (Table 2). The expression trend of *dnmt5* and *dnmt8* did not show significant differences between treated and control ovaries (Table 2).

## Discussion

This study examined whether BPA concentrations similar to those found in the environment affect female reproduction by altering endocrine signaling and/or local factors at the level of the ovary, and whether the well-established harmful effects of BPA are exerted via deregulation of epigenetic mechanisms. These working hypotheses are based on a study^6^ suggesting that even pico- and nanomolar BPA concentrations interact with the ERα or β isoforms, leading to gene expression alteration. Matsushima and colleagues found that the effect of such small concentrations relies on strong binding with estrogen-related receptor γ (ERR γ), and showed for the first time that this nuclear receptor forms a complex with BPA^19^. Moreover, BPA administration to pregnant Agouti viable yellow mouse (A^vy^) induced DNA hypomethylation in the epigenome of the offspring, demonstrating a role for it in epigenetic changes^7^.

The relationship between histone modifications and BPA has been explored only by Doherty and co-workers^20^, who documented the ability of BPA to promote histone methyltransferase EZH2 (Enhancer of Zeste Homolog 2) protein translation in mouse mammary gland exposed to 5 mg/kg BPA. Changes in EZH2 protein synthesis are usually associated with an increase in H3K27me3, which typically leads to gene repression^21^,^22^. Kundakovic and Champagne^5^ have suggested that BPA-induced gene expression, which depends on the chromatin structure, could be up- or down-regulated both by DNA methylation and histone modifications occurring in specific gene regulatory regions.

To shed some light on the issue, this study adopted a multidisciplinary approach including gene expression analysis and monitoring of histone modifications. The expression of some key genes involved in different phases of oogenesis was analyzed to elucidate the effect of BPA on the process. In particular, *star* and *cyp11a1* were selected as biomarkers of steroidogenesis. The former is involved in cholesterol transfer through the mitochondrial membrane^8^ and the latter, a member of the cytochrome P450 family, in the conversion of cholesterol to pregnenolone^23^. Indeed, given its role in early oogenesis, *cyp11a1* has proved to be a valuable biomarker of the effects of EDCs on fish^8^.

The three ER isoforms found in zebrafish ovary (*esr1*, *esr2a*, and *esr2b*) were investigated for their important role in follicle development, given the ability of EDCs to interact with genes presenting the estrogen response element (ERE) region^6^, while *fshr* was included because it is part of the hormonal cascade, which comprises the synthesis of 17β-estradiol in the gonads and of vitellogenin in the liver, that ultimately leads to oocyte growth^24^.

The signals responsible for oocyte maturation were also examined for BPA-induced effects. The most important are *lhcgr*, which is stimulated by luteinizing hormone and induces production of progesterone and its receptors (*pgrmc1* and *pgrmc2*), and *pgrmc1* and *pgrmc2*, which induce the first meiotic division, or germinal vesicle breakdown.

Even though oogenesis is mainly controlled by pituitary gonadotropins and their receptors, local factors such as *bmp15* and *gdf9* also play a large role, since they inhibit the early maturation of oocytes^25^,^26^,^27^.

Finally, the demonstration by our group^28^ that autophagy - unlike apoptosis - increases energy recycling efficiency, exerting a favorable influence on zebrafish reproduction, prompted the investigation of two biomarkers for each of these processes.

The results showed higher mRNA levels of *caspase3* and *tp53*, two markers of apoptosis, in the group exposed to 5 μg/L BPA, and a lack of effect on the autophagy process, suggesting inhibition of reproduction. The other findings suggest that the lowest BPA concentration was the most harmful, since it significantly down-regulated *esr1*, *esr2a*, *lhcgr*, and *pgrmc1*, but not *esr2b*, *star*, *cyp11a1*, and *fshr* expression.

Therefore, since the signals related to steroidogenesis and oocyte growth were not affected, it may be speculated that BPA damages oocyte maturation even at a concentration as low as 5 μg/L. These data agree both with the histology findings, which documented a greater incidence of atresia in mature oocytes from fish treated with 5 μg/L BPA compared with controls, and with the block of reproduction seen only in fish exposed to this concentration.

The results of ChIP-qPCR in zebrafish exposed to 5 μg/L BPA are also in line with the findings of molecular analyses, since enrichment of H3K4me3 and H3K27me3 histone modifications in the TSS region of the *star* and *fshr* genes decreased in the exposed groups. Their unaltered gene expression pattern may be explained by a balancing out of reduced levels of both promoter and repressor, resulting in unchanged gene transcription.

In contrast, *lhcgr*, which was down-regulated by the lowest BPA concentration, showed a different modulation of the epigenetic pattern, with a decrease and an increase, respectively, in H3K4me3 and H3K27me3 marks in its TSS region. Histone 3 lysine 4 trimethylation has been reported to promote gene transcription and specifically marks the TSS region, whereas the increment of histone 3 lysine 27 trimethylation in the promoter region often leads to repression of gene expression^29^,^30^. Therefore, *lhcrg* downregulation in fish treated with the low BPA concentration is due to an increase of H3K27me3 in the TSS region, demonstrating the adverse effect of BPA on the epigenome.

DNMTs have recently been suggested to mediate the interplay between histone modifications and DNA methylation^15^,^31^. However, both processes should thoroughly be examined to gain insights into the toxic effects of pollutants on the epigenome^32^. We therefore measured the gene expression levels of the zebrafish DNMT isoforms identified by Kamstra and colleagues^32^ in fish exposed to the low BPA concentration. For reasons that are still unclear, zebrafish have a large number of DNMTs (*dnmt1* to *8*). It has been suggested that each has a specific function - *dnmt1* would maintain methylation while *dnmt3-8*, especially *dnmt3* and *dnmt8*, would be involved in de novo methylation and share similarities with mammalian DNMT3^32^.

Moreover, a relationship has been suggested between human disease and the deregulation of epigenetic mechanisms^33^,^34^,^35^.

The above considerations suggest that upregulation of *dnmt1* and *dnmt3* in fish exposed to 5 μg/L BPA could be related to the histone modifications, further supporting the notion of BPA-induced epigenetic deregulation.

In conclusion, the lowest BPA concentration consistent with environmental exposure proved to be the most harmful, affecting *D. rerio* oocyte growth and maturation, and down-regulating the signals involved in the last phase of oogenesis.

These findings agree with previous data highlighting that EDCs do not follow the principle “the dose makes the poison”, but rather exhibit a U-shaped and inverted U-shaped, non-monotonic, dose response curve, where the strongest responses may be elicited by the lowest and highest doses, or by intermediate concentrations, respectively^36^. BPA may thus act in a U-shaped fashion, with low concentrations inducing the strongest response. The hypothesis is supported by several studies, which have suggested that such curve shapes may depend on receptor downregulation induced by higher hormone levels^37^,^38^,^39^.

Even though the present data are preliminary, and further work is needed, particularly to gain additional information on the methylation pattern of the genes involved in reproduction, these findings strongly suggest that the adverse effects of BPA on reproduction are due to its upstream ability to deregulate epigenetic mechanisms. Given the high synteny between the zebrafish and human genome, this evidence may be a starting point to explore the role of EDCs in the decline of human fertility^17^,^18^, and to shed light on the BPA-induced epigenetic changes affecting human ovary.

## Methods

### Animals and BPA administration

A total of 48 adult female zebrafish (*D. rerio*, AB wild-type strain) were placed in eight 10-L aquaria (6 fish/tank) with oxygenated water under controlled conditions (28.0 ± 0.5 °C) and maintained on a 14/10 h light/dark cycle. They were fed 4 times a day, twice commercial food (Vipagran; Sera, Loessnitz, Germany) and twice *Artemia salina*. There were a control and 3 exposed groups, which received 5, 10, or 20 μg/L BPA (98% analytical purity, Sigma-Aldrich, Milano, Italy) for 3 weeks. All tanks were maintained in duplicate.

After 3 weeks, fish were lethally anesthetized with 500 mg/l MS-222 (3-aminobenzoic acid ethyl ester, Sigma Aldrich) buffered to pH 7.4. Five half ovaries from each experimental group, in duplicate, were removed and fixed in Bouin’s solution; the remaining ovaries were used for Real Time semi-quantitative Polymerase Chain Reaction (qPCR) (5 half ovaries) and Chromatin ImmunoPrecipitation (ChIP) analysis (7 whole ovaries). All procedures complied with Italian animal experimentation laws and were approved by the Ethics Committee of Padua University (Prot. 112/2015-PR).

### Egg collection and fertility

Starting on the 8^th^ day of treatment, the 3 groups of BPA-exposed females and control females were crossed with untreated males, and fertility was determined during the following 15 days. Fertilized eggs were counted and the fertility rate was calculated as the mean ± standard deviation (SD) of fertilized egg number/female/day from the 8^th^ to the 21^th^ day of treatment.

### RNA extraction and cDNA synthesis

Total RNA was isolated from the ovary with RNAzol solution (Sigma Aldrich) according to the manufacturer’s instructions. Its final concentration was determined using Nanophotometer TM P-Class (Implen GmbH, Mϋnich, Germany), whereas integrity was established by Gel Red staining of 28S and 18S ribosomal RNA fragments on 1% agarose gel.

Total RNA was treated with DNAse to remove genomic DNA (10 IU at 37 °C for 10 min; Fermentas MBI, Amherst, NY, USA). Then 1 μg total RNA was used for cDNAs synthesis with iScript cDNA Synthesis Kit (Bio-Rad, Milano, Italy), and kept at −20 °C until use.

### Real-Time qPCR

Relative quantification of gene expression was performed with the SYBR Green method in an iQ5 Multicolor Real-Time PCR Detection system (Bio Rad). All samples were analyzed in duplicate. Reactions contained 1 μl cDNA diluted 1/10, 5 μl 2X SYBR Green PCR Master Mix (Bio Rad) containing SYBR Green as a fluorescent intercalating agent, 0.1 μM of forward and reverse primers (ED Table 3), and 3.8 μl of milliQ water. The thermal profile was as follows: enzyme activation at 95 °C for 3 min; 45 cycles of denaturation (10 sec at 95 °C) followed by 20 sec annealing at 60 °C for *star*, *dnmt6*, *esr2a*, *gdf9*, *ambra1a*, *beclin1*, *tp53*, *caspase3*, and *cyp11a1*, 59 °C for *dnmt1*, *dnmt8*, *dnmt3*, *dnmt5*, *fshr*, *pgrmc1*, *pgrmc2*, *bmp15*, and 58 °C for *dnmt4*, *dnmt7*, *esr1*, *esr2b*, and *lhcgr*, and 20 sec elongation at 72 °C. Fluorescence was monitored at the end of each cycle. Dissociation curves for primer specificity and absence of primer-dimer formation check were performed and consistently showed a single peak.

Genes *18S* rRNA^40^ and *arp (*acid ribosomal protein)^41^ were used as internal controls to enable result standardization by eliminating variations in mRNA and cDNA quantity and quality^42^. These genes were chosen because their mRNA levels did not vary either between experimental treatments or between follicular stages. No amplification product was observed in the negative control (absence of template). Data were analyzed using iQ5 Optical System version 2.1 (Bio-Rad). The quantification method was based on a ΔΔCt calculation implemented with the Pfaffl equation, to improve accuracy by accounting for varied reaction efficiencies depending on primers^43^,^44^. All results are expressed with respect to control ovaries. Data were generated in duplicate from 5 biological replicates.

### Histology

Five ovaries per fish group were fixed in Bouin’s solution and prepared for histological examination using standard biological procedures. Gonads were embedded in paraffin and sectioned (7 μm) with a microtome. Each ovary was fully sectioned, processed for hematoxylin-eosin staining, and observed at 200× final magnification under a light microscope (Leica Microsystems Inc., Milano, Italy). Atretic follicles were identified based on specific morphological markers of follicular atresia (i.e. granulosa cell hyperplasia, invagination and breakdowns of zona radiata, basal membrane disintegration, absorption of vitellus) as described by Üçüncü and Çakıcı (2009)^45^.

Images (50× final magnification) were captured using a high-resolution digital camera (DC300F; Leica Microsystems) and atretic follicle number was counted and expressed as a percentage of the number of atretic/total follicles.

Student’s t-test and ANOVA followed by Duncan’s test for multi-group comparisons were performed, as appropriate, to assess the significance of differences. Data were expressed as percentages and reported as mean values ± SD.

### Chromatin immunoprecipitation and antibodies

Chromatin was prepared from frozen ovaries following the instructions of the ChIP-IT High Sensitive Kit (Active Motif catalog no. 53040). Chromatin was fragmented by sonication with Active Motif’s EpiShearSonicator (80% amplitude, pulse for 30 sec on and 30 sec off for a total sonication “on” time of 12 min –or 22 min of elapsed time) to produce fragments ranging from 200 to 600 bp. Approximately 200 μl of chromatin was used for each immunoprecipitation reaction. Then, 50 μl was removed from each sample and used as input control. ChIP was performed using antibodies specific for H3K4me3 (Abcam) and H27K4me3 (diagenode).

### ChIP-qPCR

ChIP-qPCR was performed with real time PCR using the SYBR Green method. The reaction consisted in 5 μl SYBR Green Reaction Mix, 1 μl 0.1 μM primer pairs, 3 μl sterile water, and 1 μl DNA sample (ChIP or Input), for a total volume of 10 μl. Gene mapping and information on regions of interest were obtained using the UCSC Genome Browser. Primers were designed to target the TSS, the two flanking regions, and the gene body region using Pick Primers from the NCBI website, and were tested by *in silico* PCRs on the UCSC Genome Bioinformatics website. Primer sequences are reported in ED Table 4. For each pair, primer efficiency was tested on zebrafish genomic DNA by means of a standard qPCR curve using serial dilutions of gDNA (from 4 ng/μl to 0.004 ng/μl). Each dilution was assayed in triplicate.

qPCR analyses were performed in a 7900 HT Fast Real-Time PCR System (Applied Biosystems) using technical triplicates. The thermal protocol was as follows: initial 10-min denaturation at 95 °C, followed by 40 cycles of 95 °C for 15 sec; 60 °C for 30 sec; and 72 °C for 30 sec. ChIP-qPCR signals were calculated as percentage of input and SD was measured and represented as error bars. Data were obtained from 7 biological replicates.

### Statistical analysis

Statistical analysis of gene expression was performed with one-way ANOVA followed by Tukey’s multiple comparison test. Significance was set at *p* *<* 0.05. All procedures were performed with GraphPad Prism 6. Numerator and denominator degrees of freedom are respectively 3 and 17. F and P values were also calculated for each gene analyzed and are reported in the legends.

## Additional Information

**How to cite this article**: Santangeli, S. *et al.* Bpa-Induced Deregulation Of Epigenetic Patterns: Effects On Female Zebrafish Reproduction. *Sci. Rep.* **6**, 21982; doi: 10.1038/srep21982 (2016).

## Figures and Tables

**Table 1:** Analysis of local factors and genes involved in apoptosis and autophagy.

Gene	Ctrl	BPA 5 μg/L	BPA 10 μg/L	BPA 20 μg/L
*bmp15*	2.56 ± 0.25^a^	2.51 ± 0.94^a^	2.29 ± 0.42^a^	2.01 ± 0.65^a^
*gdf9*	2.33 ± 0.33^a^	2.50 ± 0.95^a^	2.77 ± 0.44^a^	3.31 ± 0.39^a^
*caspase3*	1.65 ± 0.45^a^	3.54 ± 0.25^b^	3.03 ± 0.47^b^	3.61 ± 0.09^b^
*tp53*	2.40 ± 0.45^a^	3.73 ± 0.61^b^	2.43 ± 1^a^	2.93 ± 0.27a,b
*ambra1a*	3.23 ± 0.54^a^	2.41 ± 0.75^a^	2.34 ± 0.81^a^	2.22 ± 0.54^a^
*beclin1*	1.43 ± 0.15^a^	1.83 ± 0.56^a^	1.9 ± 0.38^a^	1.6 ± 0.37^a^

*bmp15*: bone morphogenetic protein 15 (F = 0.7821; P = 0.5222); *gdf9*: growth differentiation factor 9 (F = 2.513; P = 0.0931); *caspase3* (F = 35.65; P = 0.0001); *tp53*: tumor protein 53 (F = 5.594; P = 0.0081); *ambra1a*: autophagy/beclin-1 regulator 1a (F = 2.336; P = 0.1101); *beclin1* (F = 1.460; P = 0.2607). Data are expressed as mean ± standard deviation; letters indicate differences between treatments (*p < *0.05 compared with untreated controls; ANOVA followed by Tukey’s multiple comparison tests). Data were generated in duplicate from five biological replicates.

**Table 2:** Transcriptional profiles of DNA (cytosine-5-)-methyltransferase genes.

Gene	Ctrl	BPA 5μg/L
*dnmt1*	1.69 ± 0.84	19.14 ± 0.9*
*dnmt3*	2.12 ± 1.04	5.41 ± 0.64*
*dnmt4*	3.86 ± 1.06	1.58 ± 0.55*
*dnmt5*	2.47 ± 1	2.82 ± 0.66
*dnmt6*	2.61 ± 0.33	1.64 ± 0.32*
*dnmt7*	5.51 ± 0.9	2.16 ± 1.15*
*dnmt8*	2.16 ± 0.63	2.18 ± 0.51

*dnmt1 to 8*: DNA (cytosine-5-)-methyltransferase genes 1 to 8. Data are expressed as mean ± standard deviation*p < 0.05 compared with untreated controls. Data were generated in duplicate from five biological replicates.

**Table 3:** Lists of primers used in gene expression analyses by Real-Time qPCR.

Gene	Forward primer (5′-3′)	Reverse primer (5′-3′)	Acc number	Gene ID
*18S rRNA*	TCGGAAAACGGTGAACCTG	AAGGTCTTTGAACCCACGG	NM_001098396.1	100037361
*arp*	CTGAACATCTCGCCCTTCTC	TAGCCGATCTGCAGACACAC	NM131580.2	58101
*star*	CCAAGTGCAGATGACCCCAA	GGAAGGTGTGTGCCCTTGTT	NM_131663.1	63999
*cyp11a1*	GCAGGATTGCCGAGACTGA	TCTGCTGGCATTCAGTGGT	AF527755.1	80374
*esr1*	GGTCCAGTGTGGTGTCCTCT	AGAAAGCTTTGCATCCCTCA	NM_152959.1	259252
*esr2a*	TAGTGGGACTTGGACCGAAC	TTCACACGACCACACTCCAT	NM_180966.2	317734
*esr2b*	TTGTGTTCTCCAGCATGAGC	CCACATATGGGGAAGGAATG	NM_174862.3	317733
*fshr*	GATTCTTCACCGTCTTCTCC	TGTAGCTGCTCAACTCAAACA	NM_001001812.1	195820
*lhcgr*	GGCGAAGGCTAGATGGCACAT	TCGCAATCTGGTTCATCAATA	NM_205625.1	402920
*pgrmc1*	CGGTTGTGATGGAGCAGATT	AGTAGCGCCAGTTCTGGTCA	NM_001007392.1	492520
*pgrmc2*	ACAACGAGCTGCTGAATGTG	ATGGGCCAGTTCAGAGTGAG	NM_213104.1	406378
*bmp15*	AGGGTGACCGGATCACTATG	TGCTGCCAGACTTTTTAGACC	NM_001020484.1	334183
*gdf9*	CGACCACAACCACCTCTCTCC	GGGACTGAGTGCTGGTGGATGCC	NM_001012383.1	497643
*caspase3*	GTGCCAGTCAACAAACAAAG	CATCTCCAACCGCTTAACG	NM_131877.3	140621
*ttp53*	GGCTCTTGCTGGGACATCAT	TGGATGGCTGAGGCTGTTCT	AF365873.1	30590
*beclin1*	GGACCACTTGGAACAACT	CCGAAGTTCTTCAGTGTCCATC	AB266448.1	393846
*ambra1a*	TCTTTCGAGAAATGGCACCT	CTCTCTGCGTTAGGGACAGG	HE602022.1	100332642
*dnmt1*	GAGCCTGTGAAGCAGGAGAA	CATGAATGGCACTGCACAGA	NM_131189.2	30430
*dnmt3*	AAACAACGCGCTTCCACG	TTCCATAACCACCACCGTCC	AF135438.1	30659
*dnmt4*	GCGTCAGAAGTATGCGAGGA	GACCTTTCCTAGCAGGGTTGA	AB196915.1	317744
*dnmt5*	GCTCCATCACATCTCAGCCC	CAAATCCGACACCGGCAAAG	XM_009296722.1	323723
*dnmt6*	AGAAAACCCATTCGCGTCCT	GTGCCCTCGTAGAGACCTTTT	AB196917.1	553189
*dnmt7*	ATCCGACATCTCTTTGCACC	GTGAAGTGAATTTGCAGAAAGC	AB196918.1	321084
*dnmt8*	GGACGTATTGTGTCCTGCT	ATCACCAAACCACATGACCC	AB196919.1	553187

*arp*: acidic ribosomal protein; *star*: steroidogenic acute regulatory protein; *cyp11a1*: cytochrome P450, family 11, subfamily A; *esr1*: estrogen receptor 1; *esr2a*: estrogen receptor 2a; *esr2b*: estrogen receptor 2b; *fshr*: follicle stimulating hormone receptor; *lhcgr*: luteinizing hormone/choriogonadotropin receptor; *pgrmc1*: progesterone membrane receptor component 1; *pgrmc2*: progesterone membrane receptor component 2; *bmp15*: bone morphogenetic protein 15; *gdf9*: growth differentiation factor 9; *tp53*: tumor protein 53; *ambra1a*: autophagy/beclin-1 regulator 1a; *dnmt1* to *8*: DNA (cytosine-5-)-methyltransferases.

**Table 4:** Sequences, location and product size of primers used in Chip-qPCR analysis.

Oligo name	Sequence 5′ to 3′	PCR product location	Product size (bp)
*fshr_1*	For: AACACCGAAGACACACTTGC Rev: CTTGTTGCCCAGACAGATGA	chr12: 26245412 + 26245492	81
*fshr_2*	For: GACACAGGAGAGTGGGTTTT Rev: ACCTGCTTGAGAAACCAGTG	chr12: 26249492 + 26249616	125
*fshr_3*	For: AATGCAGAACCATACTGACCG Rev: CTTGGACAGGCTTCTGAACT	chr12: 26264368 + 26264451	84
*fshr_4*	For: AGCCTGATTTGACTGGGTTG Rev: TGTAATGACAGCAGCCGTTT	chr12: 26272745 + 26272876	132
*fshr_5*	For: CCCTACACATAAACCGCAAGA Rev: AACATAGCCAACTGAACCCA	chr12: 26242191 + 26242307	117
*lhcgr_1*	For: CTTTCTGCTGTGGAGTGTGT Rev: TACAGTCTGCTGAGGCTCTT	chr13: 48032912 + 48033020	109
*lhcgr_2*	For: CCTTCAATAACCTGCCGAAC Rev: TTTTGTTCCAGTCTGCGGTA	chr13: 48053608 + 48053757	150
*lhcgr_3*	For: TTCAGCCTGTCTGCAATCTC Rev: GTCGATGCTACCGAAAGTGT	chr13:48076229 + 48076318	90
*lhcgr_4*	For: TCGCATGCTTATCAATGGCA Rev: TCATCGCCTCTGAAGAAACC	chr13: 48030973 + 48031116	144
*lhcgr_5*	For: ACGTGTTTACGGCCATTAGA Rev: TCCAGAGATTTGGGTGTGTG	chr13: 48037498 + 48037628	131
*star_1*	For: TCTGTAAGAAATGCCAGCACA Rev: AGGGCTATAAAGGGGCTGAA	chr8: 46837041 + 46837190	150
*star_2*	For: GTGTTCACCAGGGTCACAAT Rev: ACCAGTGCTATGTGCAACAA	chr8: 46835252 + 46835396	145
*star_3*	For: GCGTCTGCCAAATGCATAAA Rev: CCTGTATTGTCATGCGACCA	chr8: 46833862 + 46833973	112
*star_4*	For: CTCCTTCTTCGGATGTGGTT Rev: ATGATTGCCATCCACCATGA	chr8: 46836840 + 46836919	80
*star_5*	For: ACAGTGTCAGGCCATACCATA Rev: CAGTGTTGGGCTGATTACGTT	chr8: 46837645 + 46837779	135

*fshr*: follicle stimulating hormone receptor; *lhcgr*: luteinizing hormone/choriogonadotropin receptor; *star*: steroidogenic acute regulatory protein.
